# Planthoppers of Delaware (Hemiptera, Fulgoroidea), excluding Delphacidae, with species Incidence from adjacent States

**DOI:** 10.3897/zookeys.83.1176

**Published:** 2011-02-25

**Authors:** Charles R. Bartlett, Erin R. Adams, Anthony T., Jr. Gonzon

**Affiliations:** 1 Department of Entomology and Wildlife Ecology, 250 Townsend Hall, University of Delaware, Newark, Delaware, 19717-1303. U.S.A.; 2 DE Natural Heritage and Endangered Species Program, DE Division of Fish and Wildlife, DNREC 4876 Hay Point Landing Road, Smyrna, Delaware, 19977. U.S.A.

**Keywords:** Auchenorrhyncha, Fulgoromorpha, Acanaloniidae, Achilidae, Caliscelidae, Cixiidae, Derbidae, Dictyopharidae, Flatidae, Issidae, species inventory, generic key

## Abstract

The number of species of planthoppers (excluding Delphacidae) known from Delaware is updated from 7 (in 4 families) to 62 species (in 9 families). Specimen abundance is tallied by county and seasonally by two week intervals. The Chao1 abundance estimator suggests that the true fauna may be 74 species, although species incidence tallied from adjacent states (MD, NJ, PA and DC) suggests that a total fauna of approximately 100 species may be possible. An artificial key is presented to genus and select species with photos of most included taxa.

## Introduction

The distribution of planthoppers (Hemiptera: Auchenorrhyncha: Fulgoroidea) in the eastern United States was most recently summarized by Wilson and McPherson (1980a). Excluding the Delphacidae, Wilson and McPherson (1980a) reported only 4 planthopper species from Delaware, specifically Acanalonia conica (Say, 1830, Acanaloniidae), Catonia cinctifrons (Fitch, 1956, Achilidae), Melanoliarus ecologus (Caldwell, 1947; as Oliarus, Cixiidae), and Metcalfa pruinosa (Say, 1830, Flatidae). Additional species were later reported by Kramer in his revisions of the Cixiidae, specifically Cixius nervosus (Linnaeus, 1758) by Kramer (1981), Bothriocera cognita Caldwell, 1943, and Bothriocera drakei Metcalf, 1923, by Kramer (1983), bringing the reported fauna to 7 species in 4 families.

Our objectives were to provide an abundance-based list of planthopper species found in Delaware (excluding Delphacidae) established primarily on specimen records from the University of Delaware Insect Reference Collection (UDCC) in Newark, DE; provide a measure of completeness of this inventory using the Chao1 abundance-based diversity estimator (Chao 1984) and by comparison with incidence records from adjacent states (MD, NJ, PA and DC); and begin to assess their biology by providing preliminary information regarding the seasonality of the planthoppers of Delaware. We provide an artificial key to genus and select species to allow users to recognize planthopper species in the Mid-Atlantic States more easily.

## Methods

Planthopper specimens from Delaware, Maryland, New Jersey, and Pennsylvania in the UDCC were identified to species. Identification of some taxa requires dissection of male genitalia, in which case the abdomen was removed (sometimes after relaxing the specimen overnight in high humidity) and cleared for 24 hours in 15% potassium hydroxide (KOH), rinsed in water and transferred to glycerol for observation and manipulation (see, e.g., Wilson and McPherson 1980b, Bartlett and Deitz 2000). Species identification was made according to the following sources: Metcalf (1923, Derbidae except Cedusa, Dictyopharidae except Scolops and Phylloscelis, Flatidae), McAtee (1923, Derbidae: Otiocerus), Breakey (1928, Dictyopharidae: Scolops), Doering (1938, Issidae; 1939, Caliscelidae: Bruchomorpha; 1941, Caliscelidae: Aphelonema), Beirne (1950, Achilidae: Cixidia [as Epiptera]), O’Brien (1971, Achilidae: Plectoderini), Kramer (1977, Cixiidae: Oecleus; 1979, Cixiidae: Haplaxius [as Myndus]; 1981, Cixiidae: Cixius; 1983, Cixiidae: Bothriocera, Pintalia), Mead and Kramer (1982, Cixiidae: Melanoliarus [as Oliarus]), Flynn and Kramer (1983, Derbidae: Cedusa), Freund and Wilson (1995, Acanaloniidae), McPherson and Wilson (1995, Dictyopharidae: Phylloscelis). The specific identities of some taxa were confirmed by comparison with authoritatively determined specimens at the US Smithsonian Institution National Museum of Natural History (USNM), although in a few cases we examined types, or photographs of types (specifically the derbids Otiocerus signoretii Fitch, Anotia burnetii Fitch, and Anotia robertsonii Fitch from the USNM; and Otiocerus stollii Kirby and the purported type of Anotia bonnetii Kirby [but see discussion] from the Hope Entomological Collections Oxford University Museum of Natural History, OUMNH). Additional Kirby types were sought (from the British Museum, Manchester Museum, and Oxford), but are apparently missing. Females of some genera (e.g., Derbidae: Cedusa and many Cixiidae) cannot be identified to species with confidence. These specimens were tallied at the generic level and included in the specimen counts, but not included in species counts or calculation of the Chao1 statistic (see below). The artificial key to genus and select species was constructed for all taxa not requiring dissection for identification. The key was developed by modification of keys within the above listed taxonomic references. Author and year for all species is provided in table 2.

Family-level nomenclature follows Emeljanov (1999) in recognizing Acanaloniidae and Caliscelidae as independent from Issidae. Keys to families of Fulgoroidea can be found in Wilson (2005). Generic nomenclature has been updated for Cixiidae following Emeljanov (2001) and Holzinger and colleagues (2002) and for Issidae by Gnezdilov (2004).

Incidence records were listed for Maryland, New Jersey, Pennsylvania, and the District of Columbia based on literature (see below) and specimen records. Specimen records were compiled both from the UDCC and USNM collections. Specimens from Delaware were totaled by county and collection date increment. For collection date tallies, each month was divided into two increments, “early” (the 1–15^th^ of each month), and “late” (the 1^6th^-end of month) dates. Specimens with incomplete date information were omitted from these counts (resulting in the number of specimens tallied for seasonal data for some species to be less than the number of specimens observed). Because some species were at times found in abundance, seasonality records were tallied in two ways; complete specimen counts, and observation records where each series (all specimens recorded from a particular location and date) was tallied as a single observation.

To help assess completeness of the inventory, literature records were compiled from published sources (viz. Wilson and McPherson 1980a, Kramer 1981, 1983; Mead and Kramer 1982, Flynn and Kramer 1983, and McPherson and Wilson 1995) into a species incidence table. Specimen incidence records were compiled with literature records, but independently annotated.

Photographs were taken using a Nikon SMZ-1500 Digital Imaging Workstation with Nikon DS-U1 digital Camera and NIS Elements Imaging software (version 3.0). Line drawings were made by Kimberley Shropshire (see acknowledgements) by tracing photographs and rendering detail freehand with reference to specimens.

Total planthopper species richness for Delaware was also evaluated using Chao’s (1984) abundance based estimator of species richness calculated as Schao = Sobs + *F12/2F2*, where *Sobs* = # observed species, *F1* = # of species observed by exactly one specimen, *F2* = # of species observed by exactly two specimens.

## Results

Among 1,734 specimens from Delaware we observed 62 planthopper species in 27 genera and 9 families (Table 1), including 55 new state records. Not surprisingly, specimen records were strongly biased (72% of observed specimens) toward New Castle County where the main campus of University of Delaware is located. Some females in the genera Bothriocera, Cixius, Haplaxius, Melanoliarus (all Cixiidae) and Cedusa (Derbidae), representing 88 specimens, could not be definitively identified to species and these female specimens were subsequently excluded from the species tally and the calculation of the Chao1 statistic; however, one of the female Bothriocera specimens appears to represent an additional species. Specimens of Omolicna evidently represented 2 species, but we were unable to identify them or parse the species with confidence. For this reason, we have reported the specimens identified to the generic level and included them in the species count and calculations.

**Table 1.** County and seasonality records for Delaware planthoppers. Number of observed specimens given for county records, with distribution of records over the year provided, including earliest and latest observation. For seasonality records, records were divided into early (day 1–15 of the month) and late (remainder of month) observations, and for each observation a specimen count is followed parenthetically by number of independent collecting events (see methods). Sum of seasonality records may be less that sum of specimen records as ambiguous date records were omitted from seasonality tally. Column totals below seasonal entry is a count of the number of species observed during that time interval.

	County records						March		April		May		June		July		August		September		October	
	New Castle	Kent	Sussex	Sum	Early date	Late date	Early	Late	Early	Late	Early	Late	Early	Late	Early	Late	Early	Late	Early	Late	Early	Late
Acanaloniidae																						
Acanalonia bivittata	29	5	11	45	16-May	5-Oct						1(1)			5(1)	8(4)	6(5)	11(6)	9(7)	4(3)	1(1)	
Acanalonia conica	138	10	2	150	2-Jul	7-Oct									16(7)	25(17)	14(10)	24(15)	49(18)	22(8)	22(7)	2(2)
Achilidae																						
Catonia carolina	9		15	24	29-Jul	7-Oct										1(1)	4(1)	6(3)	10(1)	1(1)	1(1)	
Catonia cinctifrons	1			1	26-Jul											1(1)						
Catonia nava	10			10	3-Aug	27-Sep											1(1)			8(1)		
Catonia picta			1	1	7-Oct																1(1)	
Catonia pumila			3	3	4-Aug	8-Sep											1(1)			2(1)		
Cixidia fusca			4	4	28-Jul	7-Oct										1(1)					3(1)	
Cixidia opaca		1		1	28-Aug													1(1)				
Cixidia variegata			1	1	7-Oct																1(1)	
Caliscelidae																						
Aphelonema simplex	15	141	9	165	30-May	3-Oct						8(1)				9(1)	4(1)	69(1)		70(1)	1(1)	
Bruchomorpha sp. n.			7	7	29-Jun	21-Aug								2(1)	3(2)			2(1)				
Bruchomorpha oculata	53		1	54	22-Jun	9-Oct								4(1)	6(4)			3(2)	15(3)	12(5)	12(6)	
Bruchomorpha pallidipes			2	2	29-Jun									2(1)								
Cixiidae																						
Bothriocera cognita	9	3	34	46	22-Jun	4-Aug				1(1)				4(2)	20(5)	17(8)	2(1)					
Bothriocera drakei	2			2	29-Jun	3-Jul								1(1)	1(1)							
Bothriocera maculata			3	3	29-Jun									1(1)								
Bothriocera spp. Female	1			1	15-Jul										1(1)							
Cixius angustatus	1			1	9-May						1(1)											
Cixius nervosus	45			45	8-May	29-Jul					10(4)	1(1)		5(3)	28(6)	1(1)						
Cixius spp. Female	2			2	24-Apr	2-Jun				1(1)			1(1)									
Haplaxius ovatus	1	3		4	7-Jun	29-Jul							3(1)			1(1)						
Haplaxius pictifrons	57		3	60	18-Jun	29-Jul								27(3)	30(3)	3(3)						
Haplaxius radicus	3		1	4	11-Jun	29-Jun							3(2)	1(1)								
Haplaxius spp. Female	1			1	22-Jul											1(1)						
Melanoliarus chuliotus			1	1	12-Jul										1(1)							
Melanoliarus ecologus	90			90	16-Jun	14-Jul								66(4)	24(1)							
Melanoliarus montanus		1		1	19-Jun									1(1)								
Melanoliarus placitus	265	2	47	314	22-Apr	30-Aug				2(1)		10(3)	21(2)	38(10)	198(18)	35(12)	1(1)	3(3)				
Melanoliarus quinquelineatus	10		1	11	24-Jun	16-Jul								1(1)	9(5)	1(1)						
Melanoliarus sablensis	1			1	27-Jun									1(1)								
Melanoliarus near sablensis	6			6	16-Jun	27-Jun								6(3)								
Melanoliarus spp. Female	59		4	63	23-May	26-Jul						1(1)		47(6)	12(8)	3(3)						
Melanoliarus spp. Fem. dark wing	4	1		5	29-Jun	26-Jul								2(2)		3(3)						
Oecleus productus			2	2	11-Aug												2(1)					
Pintalia vibex	27		20	47	23-May							4(2)	3(2)	19(8)	16(5)	5(2)						
Derbidae																						
Anotia kirkaldyi	5			5	22-Jul	9-Sep										1(1)	1(1)	1(1)	2(1)			
Anotia robertsonii	5	3	1	9	19-Jun	19-Oct								1(1)		3(3)				2(2)		3(1)
Anotia westwoodi	7			7	18-Jun	1-Sep								3(2)		3(2)			1(1)			
Apache degeerii	19		10	29	1-Mar	21-Oct	9(3)								7(3)	3(3)	2(2)		1(1)	3(2)	3(1)	1(1)
Cedusa carolinensis	1			1	13-Aug												1(1)					
Cedusa near cedusa	10		1	11	26-Jul	13-Aug										6(1)	5(3)					
Cedusa kedusa	21		2	23	7-Jul	30-Aug									1(1)	19(1)	1(1)	2(1)				
Cedusa redusa	6		8	14	9-Jun	8-Sep							5(1)		5(1)		2(1)		1(1)			
Cedusa mallochi	1			1	23-Jun									1(1)								
Cedusa vulgaris	1			1	3-Aug												1(1)					
Cedusa spp. Female	16			16	9-Jun	26-Jul							1(1)		3(1)	12(1)						
Neocenchrea heidemanni	3			3	2-Sep	6-Sep													3(2)			
Omolicna spp.	11	2	23	36	14-Jun	7-Oct							1(1)		2(1)	1(1)	1(1)	17(6)	4(3)		7(1)	
Otiocerus coquebertii	1		1	2	(22-26)-June	12-Jul								1(1)	1(1)							
Otiocerus francilloni			1	1	15-Jul										1(1)							
Otiocerus reaumurii	2			2	26-Jul	9-Aug										1(1)	1(1)					
Otiocerus wolfii	7		5	12	(19-20)-July	7-Sep										2(2)	4(3)		6(2)			
Patara vanduzei	2		2	4	3-Jul	4-Aug									1(1)	2(1)	1(1)					
Sikaiana harti		1		1	1-Jul										1(1)							
Dictyopharidae																						
Rhynchomitra lingula			14	14	21-Aug	1-Sep												10(2)	4(1)			
Rhynchomitra microrhina	16	1	1	18	27-Jul	13-Sep										3(2)	4(3)	11(7)				
Scolops angustatus	8			8	18-Jul	20-Jul										2(2)						
Scolops perdix			7	7	19-Aug	21-Aug												7(2)				
Scolops pungens			1	1	17-Jul											1(1)						
Scolops sulcipes	92	2		94	11-Jul	1-Oct									1(1)	50(14)	4(4)	12(4)	3(1)	11(4)	10(1)	
Flatidae																						
Flatormenis chloris	85	18	9	112	2-Jul	21-Oct									2(1)	14(12)	16(11)	23(18)	19(12)	15(12)	8(5)	11(3)
Metcalfa pruinosa	58	12	13	83	22-Jun	11-Oct								2(2)	6(6)	15(11)	20(14)	12(7)	14(10)	5(5)	4(3)	
Ormenoides venusta	18	2	1	21	22-Jul	1-Oct										2(2)	2(2)	8(3)	5(3)		2(1)	
Fulgoridae																						
Cyrpoptus belfragei	4			4	4-Jun	18-Jul							2(2)	1(1)		1(1)						
Issidae																						
Thionia bullata	1	1		2	2-Aug	17-Oct											1(1)					1(1)
Thionia simplex	14			14	4-Jul	16-Sep									2(2)	1(1)	1(1)	2(2)	7(5)	1(1)		
Totals	1253	209	272	1734			1	0	0	3	2	6	9	24	28	36	27	19	17	13	14	5

**Figure1.**
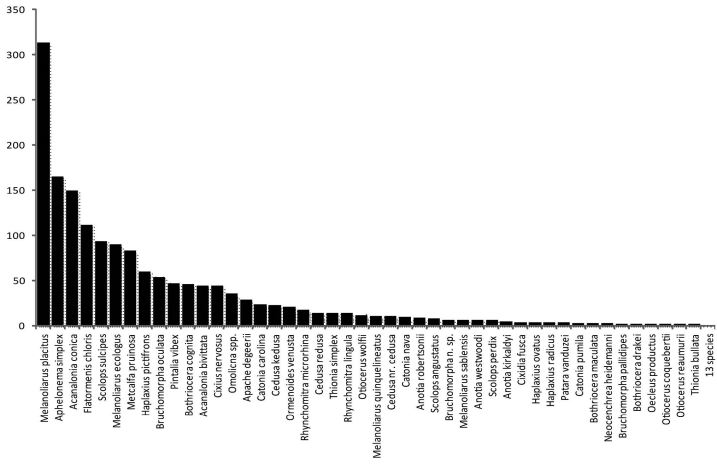
Rank abundance frequency distribution of planthopper species of Delaware. Number of specimens of each species found in Table 1.

The most abundant species were Melanoliarus placitus (18% of observed specimens), Aphelonema simplex (10%), Acanalonia conica (9%), Flatormenis chloris (7%), and Scolops sulcipes (5%), collectively representing 49% of the specimens observed (Figure 1). However, for Aphelonema simplex there were only 5 collecting events, one of which comprised 70, and a second 69 specimens (out of 165 total observed specimens). In contrast, Metcalfa pruinosa (5%) and Acanalonia bivittata (3%) were both observed in many collecting events, but these frequently encountered species are readily recognized in the field and either avoided by collectors or not accessioned by the collection manager, and therefore are probably relatively underrepresented.

The Chao1 biodiversity estimator was calculated as 74.08 species, indicating that 12 additional planthopper species are predicted to occur. The incidence list for Delaware and adjacent states (Table 2) includes 112 taxa, of which 50 species were recorded from surrounding states with no Delaware records. In addition, 22 species from MD, 5 from NJ, 8 from PA, and 21 from DC are new state records.

**Table 2.** Planthopper incidence list for Delaware, Maryland New Jersey, Pennsylvania, and the District of Columbia. Specimen records are indicated by “S”, literature records by “L”, tentative or subsequent questioned records are annotated by “?”, and records reported as erroneous by “E”. Records from Wilson and McPherson (1980) except as noted.

*Species*	*DE*	*MD*	*NJ*	*PA*	*DC*	*References and comments*
Acanaloniidae						
Acanalonia bivittata (Say, 1825)	S	S,L	S,L	S,L	S,L	
Acanalonia conica (Say, 1830)	S,L	S,L	S,L	S,L	S	
Acanalonia servillei Spinola, 1839		S		S,L		Acanalonia latifrons (Walker, 1851) synonymized with Acanalonia servillei by Fennah 1971: 334-6.
Achilidae						
Catonia carolina (Metcalf, 1923)	S	S,L	S		S	
Catonia cinctifrons (Fitch, 1856)	S,L	S,L	S,L	S,L	S	
Catonia lunata Metcalf, 1923		S,L	S,L		S	
Catonia nava (Say, 1830)	S	S,L			S	
Catonia picta Van Duzee, 1908	S		S,L			
Catonia pumila Van Duzee, 1908	S	S,L	S,L	S,L	S	
Cixidia fusca (Walker, 1852)	S	S	S,L		S	
Cixidia opaca (Say, 1830)	S	L	L	S,L		
Cixidia pallida (Say, 1830)			L	L	S,L	
Cixidia septentrionalis (Provancher, 1889)			L	L	L	
Cixidia variegata (Van Duzee, 1908)	S	S	S,L		S	
Synecdoche dimidiata (Van Duzee, 1910)		S,L	S,L	S,L		
Synecdoche grisea (Van Duzee, 1908)		S,L		L		
Synecdoche impunctata (Fitch, 1851)		S	L	L	S	
Caliscelidae						
Aphelonema decorata (Van Duzee, 1908)			L			
Aphelonema histrionica (Stål, 1864)		S				
Aphelonema rugosa (Ball, 1932)		L?				
Aphelonema simplex Uhler, 1876	S	S,L	S,L			
Bruchomorpha dorsata Fitch, 1856			S,L	L		
Bruchomorpha jocosa Stål, 1862			L		L	
Bruchomorpha oculata Newman, 1838	S	S,L	S,L	S,L	S	
Bruchomorpha pallidipes Stål, 1862	S	S	S		S,L	
Bruchomorpha sp. n.	S	S	S			
Bruchomorpha tristis Stål, 1862			L		L	
Fitchiella robertsonii (Fitch, 1856)		L		L		
Cixiidae						
Bothriocera bicornis (Fabricius, 1803)		*E*	*E*			Noted as error by Kramer 1983
Bothriocera cognita Caldwell, 1943	S,L		S,L	L		Kramer 1983
Bothriocera drakei Metcalf, 1923	S					
Bothriocera maculata Caldwell, 1943	S					
Bothriocera signoreti Stål, 1864		*E*				Noted as error by Kramer 1983
Cixius angustatus Caldwell, 1938	S	S				
Cixius apicalis Metcalf, 1923				L		Kramer 1981
Cixius coloepeum Fitch, 1856		S	L	L		
Cixius misellus Van Duzee, 1906			L	L		Kramer 1981(PA record)
Cixius nervosus (Linnaeus, 1758)	S,L	S,L	S,L	S,L		Kramer 1981
Cixius nike Kramer, 1981				S,L		Kramer 1981
Cixius pini Fitch, 1851		S,L		S,L	S	Kramer 1981
Cixius stigmatus (Say, 1825)			L	L		
Haplaxius enotatus (Van Duzee, 1909)		S,L				
Haplaxius ovatus (Ball, 1933)	S	S,L	S,L			
Haplaxius pictifrons (Stål, 1862)	S	S,L	L	S,L	S	
Haplaxius pusillus (Van Duzee, 1909)			S,L			
Haplaxius radicus (Osborn, 1903)	S	S,L			S	
Haplaxius wheeleri (Wilson, 1996)			S,L	S		
Melanoliarus chuliotus (Ball, 1934)	S				L	
Melanoliarus ecologus Caldwell, 1947	S	S,L	S,L	S,L	S,L	Mead and Kramer 1982(MD/NJ/PA)
Melanoliarus humilis (Say, 1830)	S	S,L	L	S,L	L	Mead and Kramer 1982(NJ/PA)
Melanoliarus montanus (Metcalf, 1923)	S	S,L	S	S,L		Mead and Kramer 1982
Melanoliarus placidus Van Duzee, 1912	S	S,L	S,L	S	L	Mead and Kramer 1982
Melanoliarus quinquelineatus (Say, 1830)	S	S,L	S,L	S,L		Mead and Kramer 1982
Melanoliarus sablensis (Caldwell, 1951)	S	S,L	S,L	S,L	L	Mead and Kramer 1982(MD/NJ/PA)
Melanoliarus near sablensus	S	S		S		
Melanoliarus spp. females	S	S		S		
Pentastiridius cinnamomeus (Provancher, 1889)	S		L	L		Kramer 1981
Oecleus borealis Van Duzee, 1912		S,L	S,L	S,L	S	
Oecleus productus Metcalf, 1923	S	S,L				
Pintalia delicata (Fowler, 1904)		S,L				
Pintalia vibex Kramer, 1981	S	S,L				Kramer 1981
Derbidae						
Anotia bonnetii Kirby, 1821			L			
Anotia burnetii Fitch, 1856				L		
Anotia fitchi (Van Duzee, 1893)				L		
Anotia kirkaldyi Ball, 1902	S			S,L		
Anotia robertsonii Fitch, 1856	S		L	L		
Anotia westwoodi Fitch, 1856	S	S	L	S,L	S	
Apache degeerii (Kirby, 1821)	S	S,L	S,L	S,L		
Cedusa carolinensis Flynn & Kramer, 1983	S	S,L			S	Flynn and Kramer 1983
Cedusa cedusa McAtee, 1924		S,L			S	Flynn and Kramer 1983
Cedusa chuluota Ball, 1928			S,L			Flynn and Kramer 1983
Cedusa edentula (Van Duzee, 1912)		S	L		S	Flynn and Kramer 1983
Cedusa gedusa McAtee, 1924		S,L	S,L	S,L		Flynn and Kramer 1983
Cedusa hedusa McAtee, 1924		S,L				Flynn and Kramer 1983
Cedusa incisa (Metcalf, 1923)		S		S,L		Flynn and Kramer 1983
Cedusa kedusa McAtee, 1924	S	S,L	S,L		S	Flynn and Kramer 1983
Cedusa maculata (Van Duzee, 1912)		S,L		S,L		Flynn and Kramer 1983
Cedusa mallochi McAtee, 1924	S	L				Flynn and Kramer 1983
Cedusa obscura(Ball, 1902)		S,L	S,L		S	Flynn and Kramer 1983
Cedusa redusa McAtee, 1924	S	S,L				Flynn and Kramer 1983
Cedusa shawi Flynn & Kramer, 1983		S,L				Flynn and Kramer 1983
Cedusa vulgaris (Fitch, 1851)	S	S,L				Flynn and Kramer 1983
Cedusa spp. Females	S	S		S		
Neocenchrea heidemanni (Ball, 1902)	S	L	L		L	
Omolicna uhleri (Ball, 1902)		L	L		L	
Otiocerus amyotii Fitch, 1856			L	L	L	
Otiocerus coquebertii Kirby, 1821	S	L	L	S,L		
Otiocerus francilloni Kirby, 1821	S		L			
Otiocerus reaumurii Kirby, 1821	S					
Otiocerus signoretii Fitch, 1856		S	L	L		
Otiocerus stollii Kirby, 1821		S	L	L		
Otiocerus wolfii Kirby, 1821	S	S,L	L	S,L		
Patara vanduzei Ball, 1902	S	S		L		
Shellenius ballii (McAtee, 1923)		S,L				
Shellenius schellenbergii (Kirby, 1821)			L			
Sikaiana harti (Metcalf, 1923)	S	S				
Dictyopharidae						
Mitrops dioxys (Walker, 1858)		L	L			
Phylloscelis atra Germar, 1839		L	L	S,L	L	
Phylloscelis pallescens Germar, 1839		L	L	L		
Phylloscelis rubra Ball, 1930			S,L			
Rhynchomitra lingula (Van Duzee, 1908)	S	S	S,L			
Rhynchomitra microrhina (Walker, 1851)	S	S	L	S		
Scolops angustatus Uhler, 1929	S	S,L	S,L		L	
Scolops grossus Uhler, 1876			L?			Record probably in error.
Scolops perdix Uhler, 1900	S	L	S,L	S	L	
Scolops pungens (Germar, 1830)	S	L	S,L	L	L	
Scolops sulcipes (Say, 1825)	S	S,L	S,L	S,L	L	
Flatidae						
Flatormenis chloris (Melichar, 1902)	S	S,L	S,L	S,L	L	Anormenis septentrionalis auct. (nec. Spinola, 1839) synonymized with Anormenis chloris by O’Brien 1985: 657-660, and transferred to Flatormenis by Medler 2003: 593.
Cyarda melichari Van Duzee, 1907					L	Species needs confirmation.
Metcalfa pruinosa (Say, 1830)	S,L	S,L	S,L	S,L	L	
Ormenoides venusta (Melichar, 1902)	S	S	S	S	S	
Fulgoridae						
Cyrpoptus belfragei Stål, 1869	S	L				
Poblicia fuliginosa (Olivier, 1791)		S				
Issidae						
Exortus punctiferus (Walker, 1851)			L			Originally reported by Smith (1890) as Issus aciculatus Uhler, 1876, possibly in error.
Thionia bullata (Say, 1830)	S	S	S,L	S,L	S,L	
Thionia elliptica (Germar, 1830)		S	L		L	
Thionia simplex (Germar, 1830)	S	S,L	S,L	S	L	
New records*	55	22	5	8	21	
Total species*	62	88	74	60	46	

*Unidentified females and errors excluded, Melanoliarus near sablensus included with Melanoliarus sablensus, 2 species of Omolicna counted for Delaware.

The seasonality data suggests that the optimal time of year to find planthoppers in Delaware is between late June and early August (Table 1). It appears that most species have one generation per year, although the available data is sparse for some taxa. Bruchomorpha oculata, Aphelonema simplex, and Cixius nervosus may have two generations a year. It is evident from specimens collected in logs in March that Apache degeerii overwinters as adults (early record March 1: 9 specimens from 3 collection events), although the overwintering status of other taxa is not clear from this data. Records of cixiids from late April may indicate overwintering as immatures, as has been reported for cixiids in Germany (Nickel and Remane 2002).

Specimens reported incidentally by Zuefle (2006) and Zuefle and colleagues (2008) (Table 3) provide host data for 3 Delaware planthopper species. Zuefle (2006) sampled insect use of 45 woody plants that were: 1) native, 2) non-native with native US congeners, and 3) ‘alien’ plant species with no US congeners, using pesticide knock-down or vacuum sampling. Vouchers were reported in Zuefle (2006) as ‘Oliarus sablensis’ were mostly Melanoliarus ecologus (32 of 35 dissected males were Melanoliarus ecologus and the remaining 3 Melanoliarus sablensis), so we here reported her cixiids as Melanoliarus spp. The hosts for the 3 Melanoliarus sablensis specimens were Rhododendron mucronatum, Cotoneaster lucidus, and Betula pendula. Her results confirm a polyphagous host use for Flatormenis chloris and suggest that adult Melanoliarus, or at least Melanoliarus ecologus, are polyphagous on woody plants.

**Table 3.** Planthoppers reported by Zuefle (2006) collected in Delaware by host sampled 2004–2005. Host species were segregated into 3 categories; **1** Native woody plants **2** Non-native plants congeneric with US species; and **3** “Alien” woody plants - those with no US congeners. The Melanoliarus species were reported as ‘Oliarus sablensis’, but voucher specimens in the UDCC were found to be mostly Melanoliarus ecologus with a few Melanoliarus sablensis.

Plant Family	Plant species		Planthopper species		
			Melanoliarus spp.	Flatormenis chloris	Thionia simplex
Native					
Aceraceae	Acer rubrum		0	0	0
Betulaceae	Betula nigra		0	2	0
Betulaceae	Carpinus caroliniana		2	0	0
Cornaceae	Cornus florida		0	0	0
Fagaceae	Fagus grandifolia		0	0	0
Hamamelidaceae	Hamamelis virginiana		0	0	0
Juglandaceae	Juglans nigra		4	0	0
Moraceae	Morus rubra		6	0	0
Rosaceae	Prunus serotina		0	2	0
Ericaceae	Rhododendron periclymenoides		7	0	0
Rosaceae	Rosa carolina		2	0	0
Salicaceae	Salix nigra		1	1	1
Tiliaceae	Tilia americana		1	0	0
Ulmaceae	Ulmus americana		0	0	0
Caprifoliaceae	Viburnum dentatum		8	0	0
	Subtotal		31	5	1
Non-native congeneric plants					
Aceraceae		Acer platanoides	0	2	0
Betulaceae		Betula pendula	3	0	0
Betulaceae		Carpinus betulus	0	0	0
Cornaceae		Cornus kousa	2	0	0
Fagaceae		Fagus sylvatica	3	0	0
Hamamelidaceae		Hamamelis mollis	2	0	0
Juglandaceae		Juglans regia	2	0	0
Moraceae		Morus alba	0	0	0
Rosaceae		Prunus serrulata	1	1	0
Ericaceae		Rhododendron mucronatum	42	0	0
Rosaceae		Rosa multiflora	7	1	0
Salicaceae		Salix babylonica	0	0	0
Tiliaceae		Tilia cordata	2	0	0
Ulmaceae		Ulmus parvifolia	2	5	0
Caprifoliaceae		Viburnum dilatatum	14	2	0
		Subtotal	80	11	0
Alien plants					
Lardizabalaceae		Akebia quinata	9	0	0
Fabaceae		Albizia julibrissin	5	1	0
Rosaceae		Cotoneaster lucidus	16	2	0
Fabaceae		Cytisus scoparius	9	1	0
Oleaceae		Forsythia suspensa	10	0	0
Ginkgoaceae		Ginkgo biloba	1	0	0
Araliaceae		Hedera helix	6	0	0
Sapindaceae		Koelreuteria paniculata	1	0	0
Lythraceae		Lagerstroemia indica	5	0	0
Oleaceae		Ligustrum vulgare	6	1	0
Scrophulariaceae		Paulownia tomentosa	1	0	0
Rutaceae		Phellodendron amurens	0	0	0
Rutaceae		Poncirus trifoliata	1	0	0
Rosaceae		Pyrus pashia	4	0	0
Oleaceae		Syringa vulgaris	1	0	1
		Subtotal	75	5	1
		Total	186	21	2

## Systematics

Artificial key to genus and select planthopper species from Delaware and vicinity.

**Table:** 

1	Hind tibiae with large movable spur at apex (Fig. 2A)	Delphacidae
–	Hind tibiae without movable spur at apex (e.g., Figs 2B–D)	2
2	Second tarsomere of hind legs with row of apical spines (Fig. 2E)	3
–	Second tarsomere of hind legs with one apical spine on each side (Fig. 2F) or spines absent	7
3	Larger species, greater than 10 mm, with patterned forewings (Figs 3H, I); hindwings with numerous cross veins near apex and in anal area; uncommon in study area	Fulgoridae, 71
–	Mostly smaller species, forewings variable; hindwings without cross veins near apex or in anal area	4
4	Forewings overlapping posteriorly (Figs 4G–L, 5F–L, 6F–H), trailing margins angled; body flattened	Achilidae, 13
–	Forewings not overlapping posteriorly; body variable	5
5	Beak with apical segment subequal in length and width (except Cedusa); forewings often with tubercles on claval veins (Figs 8G, 9B); antennae may bear projections (Figs 10E, F) or subtended by a shelf-like structure (Figs 10A–D); median carina of frons often absent; parameres of male much longer than pygofer	Derbidae (most), 41
–	Beak with apical segment longer than wide; forewings without tubercles on claval veins (or with tubercles on all veins); antennae never bearing projections or subtended by a shelf-like structure; median carina of frons present; parameres of male shorter than length of pygofer	6
6	Frons with two or three median carinae and/or head with elongate anterior projection (Figs 13–14); median ocellus absent; wing vein tubercles usually absent	Dictyopharidae, 60
–	Frons with one median carina; head not elongate; median ocellus usually present above frontoclypeal suture (Figs 6D–E, 7B, H); usually with tubercles on veins of wings	Cixiidae, 35
7	Forewings with tubercles on claval veins (e.g., Figs 8G, 9B), if tubercles present in claval area (Figs 3D–G) then forewings waxy with row of many small peripheral cells; beak with apical segment subequal in length and width; frons often compressed with median carina absent (Figs 10B, D, G); parameres much longer than pygofer	Derbidae (few), 41
–	Forewings without tubercles on claval veins (or with tubercles on all veins); beak with apical segment longer than wide; frons not compressed, median carina generally present (e.g., Figs 12, 15A–D); parameres shorter than length of pygofer	8
8	Forewings waxy, bearing tubercles between veins on clavus (Figs 3D–G) and with numerous costal crossveins	Flatidae, 68
–	Forewings not waxy, without tubercles on clavus; without numerous costal crossveins (e.g., Figs 3A–C, 15E–H)	9
9	Hind tibiae without lateral spines (Fig. 2B); forewings with reticulate venation, usually extending to apex of abdomen (even in brachypters); usually green (occasionally pink) (Figs 3A–C)	Acanaloniidae, 11
–	Hind tibiae with lateral spines (Figs 2C–D); forewing venation not reticulate (Figs 15E–H), brachypters may have forewings short (Fig. 11), exposing several segments in dorsal view; color not green, usually brown, black, or straw (pinkish in males of 1 species)	10
10	Usually brachypterous with forewings shorter than abdomen (Fig. 11); frons with sublateral carinae bordering a large disc-like or elongate areolet, sublateral carinae of frons meeting ventrally (or nearly so) (Fig. 12); hind tibiae with single lateral spine (Fig. 2C)	Caliscelidae, 26
–	Forewings covering abdomen (both brachypters and macropters) (Figs 15E–H); frons with median carina, with or without sublateral carinae; if present, not meeting ventrally (Figs 15A–D); hind tibiae with two lateral spines (Fig. 2D)	Issidae, 72
Acanaloniidae		
11	Body green (rarely pink) with conspicuous brownish to reddish marking along lateral portions of thoracic nota (Fig. 3B), continuing onto wings	Acanalonia bivittata
–	Body uniformly green (rarely pink) (Figs 3A, C); may have middorsal vitta on thorax	12
12	Head distinctly produced conically (Fig. 3A); without prominent median carina across vertex and thorax; abundant in Mid-Atlantic states	Acanalonia conica
–	Head not produced conically (Fig. 3C); with prominent median carina across vertex and thorax; southeastern species occasional in Mid-Atlantic States	Acanalonia servillei
Achilidae		
13	Head, including eyes, less than 2/3 as wide as pronotum (Figs 5F–J) (Myconini)	Cixidia, 14
–	Head including eyes at least 2/3 as wide as pronotum (Figs 4G–L, 6F–H) (Plectoderini)	18
14	Clypeus and upper half of frons dark brown or black, strongly contrasting with pale lower half of frons (Fig. 5B)	Cixidia opaca
–	Frons more uniformly colored, upper half not strongly contrasting (Figs 5A, C–E)	15
15	Vertex short, projecting in front of eye for distance less than length of eye (Fig. 5J); frons distinctly and uniformly speckled (Fig. 5E)	Cixidia variegata
–	Vertex elongate, projecting in front of eye for distance equal to or greater to length of eye; frons more uniformly colored (Figs 5A, C)	16
16	Frons and clypeus uniformly colored (Fig. 5D)	Cixidia septentrionalis
–	Clypeus distinctly darker than frons (Figs 5A, C)	17
17	Vertex projected in front of eye for distance greater than eye length, vertex 1.3–1.5× as long as basal width (Fig. 5F); frons and clypeus about as dark as pronotum; forewings nearly uniform brown	Cixidia fusca
–	Vertex projected in front of eye for distance about equal to eye length, vertex length about equal (1–0.95x) to basal width (Fig. 5H); frons and clypeus paler than pronotum; forewing variegated with grayish white	Cixidia pallida
18	Subcostal cell of forewing longer than 1/3 length of forewing, narrow throughout (Fig. 16B); medioventral lobe of male pygofer entire (Fig. 16G)	Synecdoche, 19
–	Subcostal cell of forewing about 1/3 length of forewing, wider before its apex (Fig. 16A); medioventral lobe of male pygofer apically bifurcate (Fig. 16F)	Catonia, 21
19	Frons entirely pale (Fig. 6B)	Synecdoche grisea
–	Frons with dark transverse bands or all dark (Figs 6A, C)	20
20	Frons with dark bands (Fig. 6C)	Synecdoche impunctata
–	Frons uniformly dark, contrasting with pale clypeus (Fig. 6A)	Synecdoche dimidiata
21	Upper dark band of frons mottled, distinctly paler than lower band (Fig. 4D); larger species usually more than 5.8 mm	Catonia nava
–	Frons, if banded (Figs 4A–C, E), with upper dark band not mottled and not paler than lower, or frons not dark banded (Fig. 4F); size less than 6.2 mm	22
22	Frons with two very dark transverse bands (Figs 4B, E)	23
–	Frons pale with pale bands, or uniformly pale (Figs 4A, C, F)	24
23	Lower dark band distinctly paler near frontoclypeal suture giving frons a tricolored appearance (Fig. 4E); body often with orangish cast	Catonia picta
–	Lower dark band uniformly dark (Fig. 4B); body brown or grayish	Catonia cinctifrons
24	Pale transverse marking at frontoclypeal suture not reaching lateral margin of frons (Figs 4A, C)	25
–	Frons uniformly colored or pale transverse marking at frontoclypeal suture extending to lateral margin of frons (Fig. 4F)	Catonia pumila
25	Pale transverse marking at level of ocelli complete, reaching lateral margin of frons (Fig. 4C)	Catonia lunata
–	Pale transverse marking at level of ocelli incomplete, not reaching lateral margin of frons (Fig. 4A)	Catonia carolina
Caliscelidae		
26	Head produced into weevil-like snout (Figs 11E–H); usually black	30
–	Head not produced (Fig. 11A–D); paler	Aphelonema, 27
27	Vertex very broad, width at least 5–6× median length (Figs 12A, D); frons greatly exposed above, fastigium rounded when viewed laterally; mostly straw to pink colored (Figs 11A, D), may have darker wings and abdomen	28
–	Vertex longer, width 2–3× median length, frons not as exposed from above (Figs 12B–C); fastigium angled when viewed laterally; mostly black and pale colored (Figs 11B–C)	29
28	Head and thorax orange-tan, rest of dorsum blackish brown (Fig. 11A, especially in males); central frontal tablet of frons pointed below (Fig. 12A); found mostly in the southeast, reported from NJ	*phelonema decorata*
–	Uniformly pale ochreous (females) to pink (most males) in color (Fig. 11D); central frontal tablet of frons almost circular (Fig 12D)	Aphelonema simplex
29	When viewed from the side, fastigium of head produced forward, frons slanted; vertex somewhat triangular (Fig. 11B)	Aphelonema histrionica
–	When viewed from the side, fastigium not produced, frons not slanted; vertex broadly rounded anteriorly (Fig. 11C)	Aphelonema rugosa
30	Middle and front tibiae expanded	Fitchiella robertsonii
–	Middle and front tibiae not expanded	Bruchomorpha, 31
31	Dorsal light stripe broad and conspicuous, extending from near apex of face to apex of forewings or beyond	Bruchomorpha sp. n.
–	Dorsal light stripe not broad and conspicuous, generally of lesser extent	32
32	Nasal process distinctly pronounced, head concave ventrally in lateral view (Fig. 11F); in dorsal view extending anteriorly beyond eye for a distance equal or greater than length of eye	Bruchomorpha oculata
–	Nasal process less pronounced, head weakly convex ventrally; in dorsal view extending anteriorly beyond eye for a distance less than length of eye (Figs 11E, G–H)	33
33	Reddish-brown in color with a dark spot on clypeus	*ruchomorpha jocosa*
–	Uniformly black, usually with light stripe on vertex (sometimes reaching thorax)	34
34	Legs pale (Fig 11G); small species, less than 2.6 mm	Bruchomorpha pallidipes
–	Legs dark (Fig 11H); large species, more than 2.6 mm	Bruchomorpha tristis
Cixiidae		
35	Antennae arising from elongated cup-like cavities anterior to eyes (Fig. 7A)	Bothriocera
–	Antennae not within cup-like cavities, arising below eyes (Fig. 7E, 7I)	36
36	Hind tibiae without spines (similar to Fig. 2B)	37
–	Hind tibiae with one or more spines along axis before apex (similar to Figs 2C–D)	38
37	Mesonotum with 5 carinae; crown strongly narrowed (Fig. 6J)	Oecleus
–	Mesonotum with 3 carinae; crown slightly narrowed (Fig. 6I)	Haplaxius
38	Mesonotum with 5 longitudinal carinae (although intermediate pair sometimes obsolete); posterior margin of crown angularly incised (Figs 7F–G)	39
–	Mesonotum with 3 carinae; posterior margin of crown quadrately or roundly incised (Figs 7D, J)	40
39	Apex of basitarsus of hind leg with 12 teeth	Pentastiridius
–	Apex of basitarsus of hind leg with no more than 10 teeth	Melanoliarus
40	Forewings roof-like in position with distal portions clearly separated (Fig. 7D); spines on hind tibiae conspicuous	Cixius
–	Forewings vertical in position with distal portions oppressed (Figs 7I–J); spines on hind tibiae inconspicuous	Pintalia
Derbidae		
41	Clavus open (Figs 16C–D; combined anal veins reaching posterior cubitus and usually curved to follow wing margin); most taxa with head projecting well beyond eyes in lateral view (e.g.,Figs 10E–F); frons very narrow (Fig. 10G); forewings twice as long as body or more, delicate appearing (Otiocerinae: Otiocerini and Sikaianini)	42
–	Clavus closed (Fig. 16E; combined anal veins reaching wing margin within claval area); most taxa with head projecting only slightly beyond eyes (Figs 10A, C); frons usually not as narrow (Figs 10B, D) (except Patara, see Fig. 8G); forewings not as long, most taxa less delicate (Otiocerinae: Patarini; Cedusinae; and Derbinae: Cenchreini)	57
42	Antennae with 2 or 3 conspicuous appendages (Figs 10E–F)	43
–	Antennae lacking appendages (Figs 8J, 10G)	51
43	General color uniformly rose or reddish (Fig. 8E); head in lateral view with vertex distinctly concave in apical third and apex pointed (Fig. 10F); dorsal margin of wings in repose sharply angled upward in apical third; forewings with dusky spots in cells	Apache degeerii
–	General color white or yellow (e.g., Figs 9D–J), although red markings may be present; head in lateral view with vertex rounded (Fig. 9I, J, 16H, I), or nearly flat (Fig. 10E); dorsal margin of wings straight or curved slightly upward	44
44	In lateral view, demarcation between vertex and frons obtusely angular (Fig. 10E)	Otiocerus, 46
–	In lateral view, demarcation between vertex and frons smoothly rounded (Figs 9I, J; 16H–I)	Shellenius, 45
45	Head in lateral view 1.5× as long as broad (Figs 9J, 16I); forewing brownish apically in trailing portion of wing; red markings reduced or absent	Shellenius schellenbergii

–	Head in lateral view 2.0× as long as broad (Figs 9I, 16H); forewings with very pale brown markings widely distributed; with red markings on head and wing	Shellenius balli
46	Wings with conspicuous round dusky spots in cells (Figs 9E, F, H)	47
–	Wings without conspicuous round dusky spots in cells (Figs 9D, G)	50
47	Apical margin of forewings with a row of spots in the cells (Figs 9E, H)	48
–	Spots not in row within apical cells (Fig. 9F)	49
48	Apex of head with a black line laterally followed by a broader red line (Fig. 9H); forewings with spots throughout	Otiocerus wolfii
–	Apex of head without a black line laterally (Fig. 9E); forewings with spots mostly in proximal half	Otiocerus francilloni
49	Forewings with a large black spot on the sutural margin (in the clavus) and four smaller ones in a square, including 1 in costal cell	Otiocerus signoretii
–	Forewings with spots arranged differently from above (Fig. 9F)	Otiocerus reaumurii
50	Color of the wings dark, without distinct band (Fig. 9G)	Otiocerus stollii
–	Color of the wings pale with distinct reddish forked band (Fig. 9D)	Otiocerus coquebertii
51	In lateral view, head projecting in front of eyes for a distance of less than half width of eyes; forewings with scattered spots	Sikaiana harti
–	In lateral view, head projecting in front of eyes for a distance subequal to width of eyes (Fig. 8J); color mostly following veins	Anotia, 52
52	Costa narrow; forewings with veins not crowded together to give appearance of a stigma (Figs 8A–D); some or most veins of forewings with smoky borders	53
–	Costa broader; Sc and R vein tips crowded together to give appearance of a stigma (Fig. 8I); forewings more extensively marked with fuscous	Anotia fitchi
53	First 3 segments of abdomen with middorsal black stripe	Anotia burnetii
–	Abdomen without middorsal black stripe	54
54	Forewings mostly pale with a few fuscous marked crossveins (Fig. 8C); apex of forewing without dark round spots	Anotia robertsonii
–	Forewings more extensively marked; most veins with smoky borders (Figs 8B, D); apex of forewing often with dark round spots	55
55	Head with a single marking, below antennae; apical border of forewings with four dark round spots in the cells (Fig. 8A)	Anotia bonnetii
–	Head with dark or red markings above and below antennae; apical border of forewings usually without round spots in the cells	56
56	At least some veins dark in color (Fig. 8B)	Anotia kirkaldyi
–	All veins pale (Fig. 8D)	Anotia westwoodi
57	Antennae terete, subtended by flattened subantennal process from gena or anterior portion of lateral margin of pronotum (Figs 10A–D), often strongly modified into a reversed “c” (in lateral view) directly behind antennae, or strongly keeled; face not strongly compressed, frons evident; clavus at least half as long as whole forewing (Derbinae: Cenchreini, and Cedusinae)	58
–	Second segment of antennae flattened (more evident in males than females), antennae not subtended by process; lateral margin of pronotum not strongly modified; face strongly compressed, frons keel-like (similar to Fig. 10G); clavus less than half as long as whole forewing (Fig. 9C) (Otiocerinae: Patarini)	Patara vanduzei
58	Subantennal process large, extending from gena, completely subtending antennae as a shelf (Fig. 10A); reduced (or absent) sensory pits on head and wings; color uniform, near black or deep grey (Fig. 9A), infrequently near white with yellowish brown patches (Cedusinae)	Cedusa
–	Subantennal process extending from pronotum, smaller (Fig. 10C); lateral carinae of vertex and second claval vein with sensory pits; color usually orange to pale (Figs 8F, 9B) (Derbinae: Cenchreini)	59
59	Media with more than two branches, connected to cubitus by crossvein; size less than 6 mm, usually distinctly orangish (Fig. 9B)	Omolicna
–	Media and cubitus each with two branches, not connected by crossveins; size over 7 mm; color orangish white (Fig. 8F)	Neocenchrea heidemanni
Dictyopharidae		
60	Head projected in front of eyes (Figs 13, 14G, H); front femora not foliaceous	63
–	Head not projected in front of eyes (Figs 14A–F); front femora foliaceous	Phylloscelis, 61
61	Eight or fewer longitudinal veins on the forewing; color either uniformly black to dark brown in dorsal view or yellowish body with reddish-brown forewings with prominent yellow wing veins (Figs 14C, F); carinae of frons indistinct	Phylloscelis atra
–	With more than 8 longitudinal veins; color not as above; carinae of frons distinct (Figs 14A–B, D–E)	62
62	Veins concolorous with forewings; body black to light reddish brown (Fig. 14D)	Phylloscelis rubra
–	Veins of forewings dark mottled with pale; body light grey-brown (Fig. 14E)	Phylloscelis pallescens
63	Forewings clear, macropterous; head projection anterior to eyes subequal in width to vertex; body green (Figs 14G–H)	Rhynchomitra, 64
–	Forewings patterned, usually brachypterous; head projection anterior to eyes narrower than vertex; body brownish (Fig. 13)	Scolops, 65
64	Head projection long (Fig. 14G), in dorsal view narrowing anterior to eyes, projected in front of eyes greater than width of vertex; upcurved in lateral view	Rhynchomitra microrhina
–	Head projection short (Fig. 14H), in dorsal view rather quadrate, projected in front of eyes for distance about width of vertex; not distinctly upcurved in lateral view	Rhynchomitra lingula
65	Costal cell of forewing with costal vein and membrane white (Fig. 13A)	Scolops angustatus
–	Costal cell of forewing with costal vein variegated (Figs 13B–D)	66
66	Forewings reticulate over apical half (especially brachypters), veins margined with dark (Figs 13D, H)	Scolops sulcipes
–	Forewings not reticulate over apical half (Figs 13B–C, F–G)	67
67	Pronotum and usually vertex with dark markings (Fig. 13G); body with grayish cast	Scolops perdix
–	Pronotum and vertex without dark markings (Fig. 13F); body with brownish cast	Scolops pungens
Flatidae
68	Wings much longer than wide, distinctly narrowing caudally to caudal apex (Fig. 3E); brown	Cyarda
–	Wings slightly longer than wide, truncate to broadly rounded caudally (Figs 3D, F–G); green or grey	69
69	Body grey to blackish (Fig. 3F); forewings with single row of marginal cells along apical and trailing margin (set off by a submarginal vein)	Metcalfa pruinosa
–	Body green (Figs 3D, G); forewings with one or two rows of marginal cells	70
70	Frons broader than long; forewings with two rows of marginal cells along apical and trailing margin (set off by two submarginal veins) (Fig. 3D); wings usually rather truncate apically	Flatormenis chloris
–	Frons longer than broad; forewings with one row of marginal cells (Fig. 3G); wings usually rounded apically (forewings often with orangish cast along apices)	Ormenoides venusta
Fulgoridae
71	Forewings and much of body nearly black (Fig. 3I); caudal abdominal tergites red; head in lateral view with frons at acute angle from vertex; flange of head behind eye small	Poblicia fuliginosa
–	Forewings and body mottled (Fig. 3H), predominately reddish brown; abdomen not red; head in lateral view with frons at sharp angle from vertex; flange of head behind eye distinct	Cyrpoptus belfragei
Issidae
72	Hind wings absent or rudimentary; smaller insects, less than 4.5 mm (Figs 15A, E); southeastern species, reported from NJ, possibly in error	Exortus punctiferus
–	Hind wings present, entire, with strongly marked notches at the joints of the folds, anal area large; larger insects varying from 5.5 to 8.0 mm (Figs 15B–D, F–H)	Thionia, 73
73	Uniformly colored, lacking proximal bulla (Fig. 15H); carinae of face weak (Fig. 15D)	Thionia simplex
–	Body patterned, wings with proximal bulla (Figs 15F–G); carinae of face conspicuous (Figs 15B–C)	74
74	Vertex broader than long; distinctly concave in frontal view with lateral margins elevated (Fig. 15B)	Thionia elliptica
–	Vertex longer than broad, slightly concave in frontal view, lateral margins not strongly elevated (Fig. 15C)	Thionia bullata

**Figure 2.**
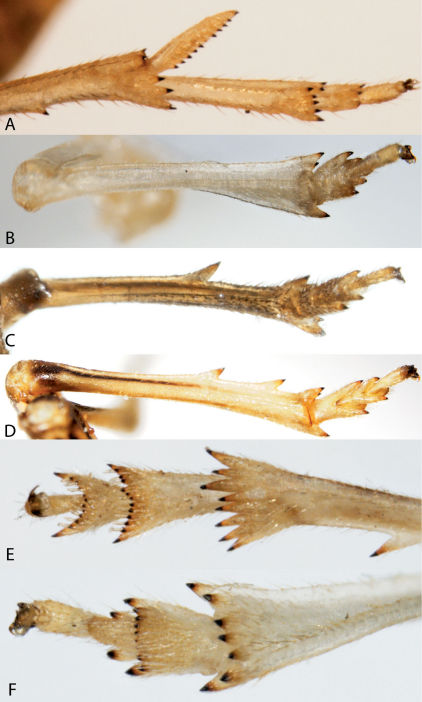
Hind legs of planthoppers. **A** Delphacidae, tibia with calcar **B** Acanaloniidae, tibia without spines **C** Caliscelidae, tibia with 1 spine **D** Issidae, tibia with 2 spines **E** Dictyopharidae, second tarsal segment with row of teeth **F** Acanaloniidae, second tarsal segment with pair of spines.

**Figure 3.**
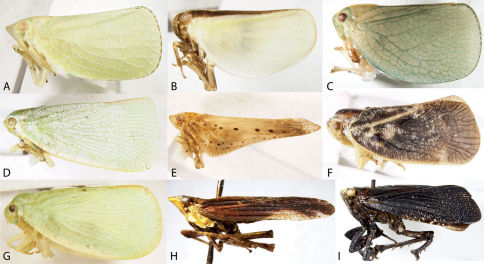
Lateral habitus of Acanaloniidae, Flatidae, and Fulgoridae. **A** Acanalonia conica **B** Acanalonia bivittata **C** Acanalonia servillei **D** Flatormenis chloris **E** Cyarda sp. **F** Metcalfa pruinosa **G** Ormenoides venusta **H** Cyrpoptus belfragei **I** Poblicia fuliginosa.

**Figure 4.**
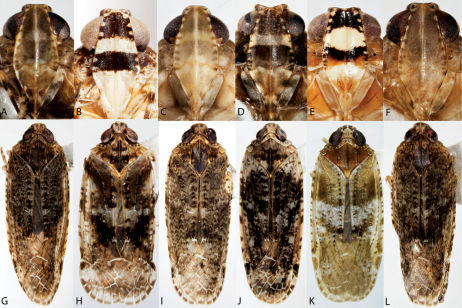
Habitus of Catonia (Achilidae) (**A–F** frons, **G–K** dorsal view). **A,** **G** Catonia carolina **B,** **H** Catonia cinctifrons **C,** **I** Catonia lunata **D,** **J** Catonia nava **E,** **K** Catonia picta **F,** **L** Catonia pumila.

**Figure 5.**
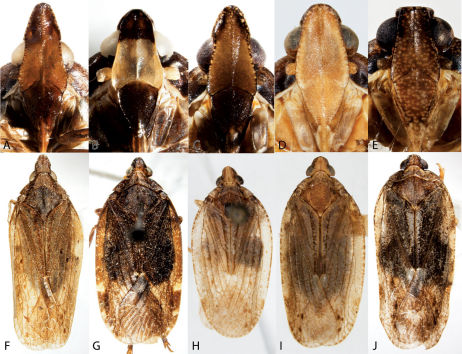
Habitus of Cixidia (Achilidae) (**A–E** frons, **F–J** dorsal view). **A,** **F** Cixidia fusca **B,** **G** Cixidia opaca **C,** **H** Cixidiapallida **D,** **I** Cixidia septentrionalis **E,** **J** Cixidia variegata.

**Figure 6.**
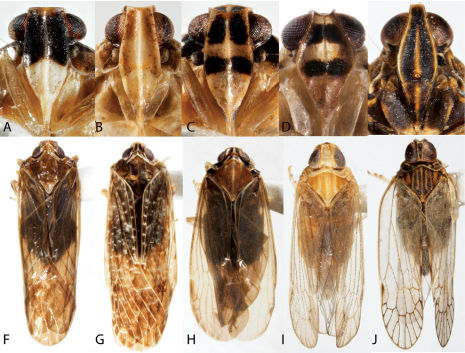
Habitus of Synecdoche (Achilidae), Haplaxius and Oecleus (Cixiidae) (**A–F** frons, **F–J** dorsal view). **A,** **F** Synecdoche dimidiata **B,** **G** Synecdoche grisea **C,** **H** Synecdoche impunctata **D,** **I** Haplaxius pictifrons **E,** **I** Oecleus borealis.

**Figure 7.**
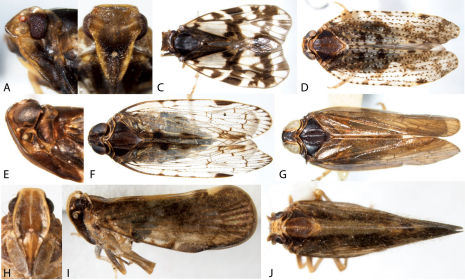
Habitus of Cixiidae. **A** Bothriocera cognita, head, lateral view **B** same, frons **C** same, dorsal view **D** Cixius pini, dorsal view **E** Melanoliarus placidus, head, lateral view **F** same, dorsal view **G** Pentastiridius cinnamomeus, dorsal view **H** Pintalia vibex, frons **I** same, lateral view **J** same, dorsal view.

**Figure 8.**
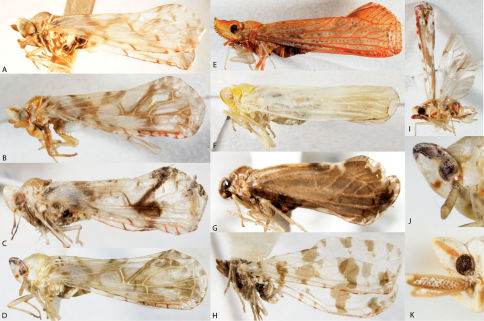
Lateral habitus of Derbidae I. **A** Anotia bonnetii **B** Anotia kirkaldyi **C** Anotia robertsonii **D** Anotia westwoodi **E** Apache degeerii **F** Neocenchrea heidemanni **G** Patara vanduzei **H** Sikaiana harti **I** Anotia fitchi **J** Anotia westwoodi, head lateral view; **K** Sayiana sayi, head lateral view.

**Figure 9.**
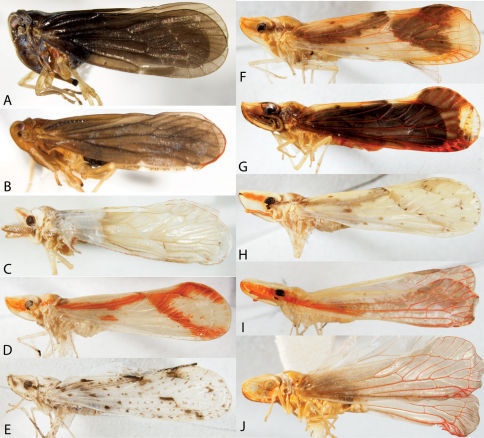
Lateral habitus of Derbidae II. **A** Cedusa sp. **B** Omolicna sp. **C** Sayiana sayi **D** Otiocerus coquebertii **E** Omolicna francilloni **F** Omolicna reaumurii **G** Omolicna stollii **H** Omolicna wolfii **I** Shellenius balli **J** Shellenius chellenbergii.

**Figure 10.**
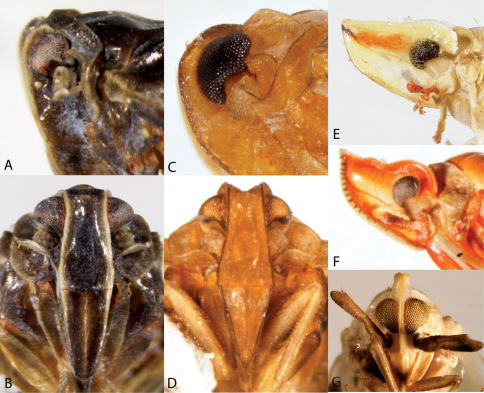
Heads of Derbidae. **A** Cedusa sp. lateral view **B** Cedusa sp., frontal view **C** Omolicna sp., lateral view **D** Omolicna sp., frontal view **E** Otiocerus wolfi, lateral view **F** Apache degeerii, lateral view **G** Anotia robertsonii, frontal view.

**Figure 11.**
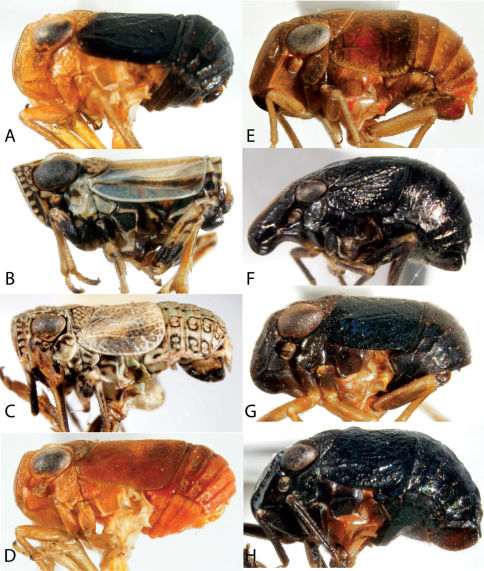
Lateral view of Caliscelidae. **A** Aphelonema decorata **B** *phelonema histrionica* **C** *phelonema rugosa* **D** *phelonema simplex* **E** Bruchomorpha jocosa **F** Bruchomorpha oculata **G** Bruchomorpha pallidipes **H** Bruchomorpha tristis.

**Figure 12.**
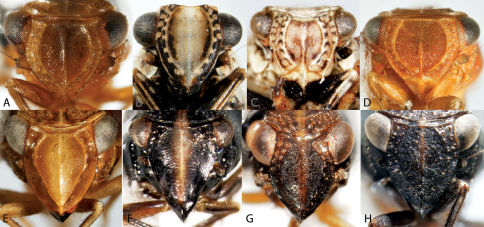
Frontal view of Caliscelidae. **A** Aphelonema decorata **B** Aphelonemahistrionica **C** Aphelonemarugosa **D** Aphelonemasimplex **E** Bruchomorpha jocosa **F** Bruchomorpha oculata **G** Bruchomorpha pallidipes **H** Bruchomorpha tristis.

**Figure 13.**
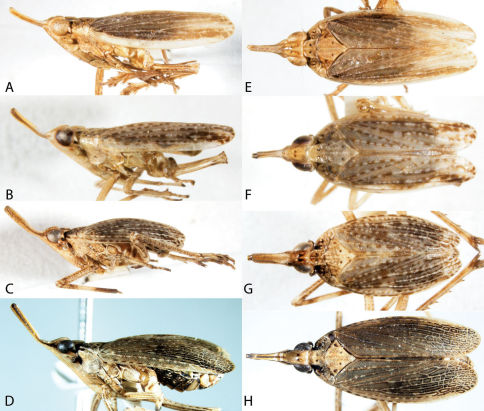
Habitus of Scolops (Dictyopharidae). **A, E** Scolops angustatus **B, F** Scolops perdix **C, G** Scolops pungens **D, H** Scolops sulcipes.

**Figure 14.**
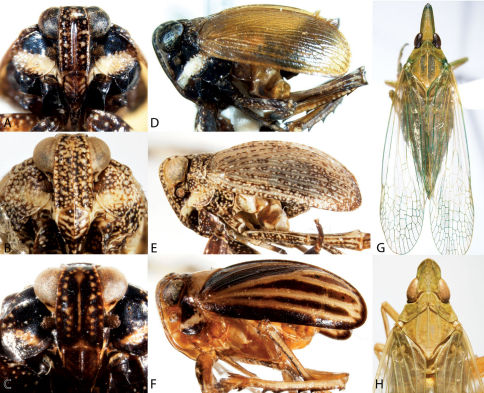
Phylloscelis and Rhynchomitra (Dictyopharidae) (**A–C, G** Dorsal view habitus, **D–F** frontal view, **H** Dorsal view, head and thorax). **A, D** Phylloscelis rubra **B, E** Phylloscelis pallescens **C, F** Phylloscelis atra **G** Rhynchomitra microrhina **H** Rhynchomitra lingula.

**Figure 15.**
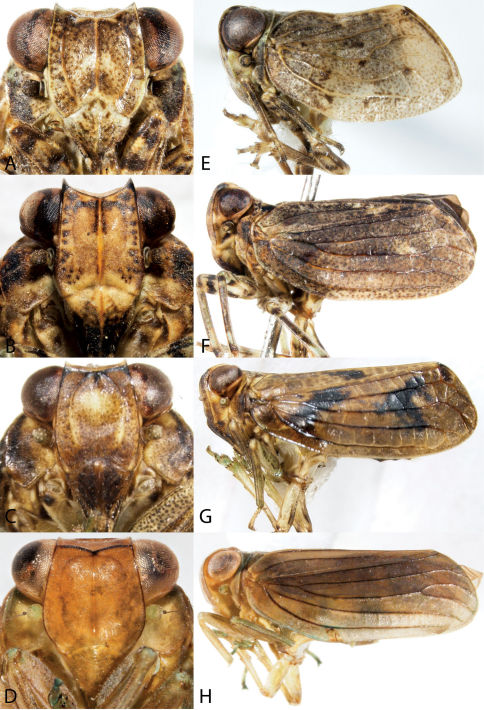
Issidae (**A–D** frontal view **E–H** Lateral view). **A, E** Exortus punctiferus **B, F** Thionia bullata **C, G** Thionia elliptica **D, H** Thionia simplex.

**Figure 16.**
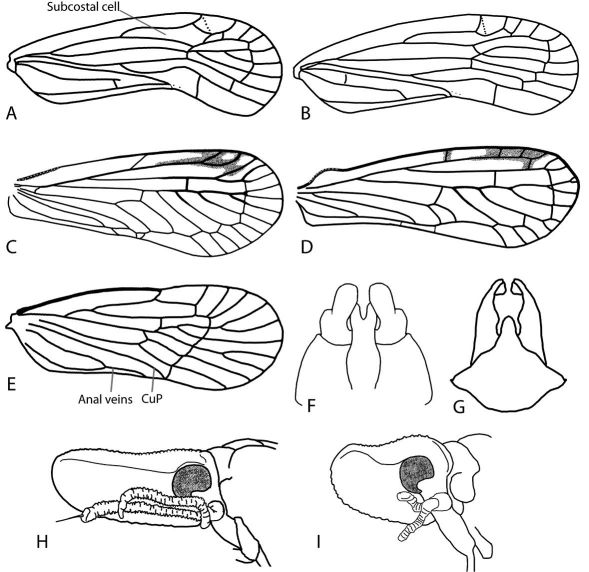
Line drawings of Achilidae and Derbidae (**A–E** Forewing, head left, costal margin top **F–G** Male genitalia, ventral view **H–I** Shellenius spp., head, left lateral view). **A** Catonia pumila; **B** Synecdoche rubella (Van Duzee, 1910); **C** Anotia fitchi; **D** Anotia robertsonii; **E** Cedusa sp.; **F** Catonia picta; **G **Synecdoche dimidiata; **H** Shellenius balli; **I** Shellenius schellenbergii (**A–B** Redrawn from O’Brien, 1971; **E** redrawn from Metcalf, 1923).

## Discussion

### Biodiversity

This survey brings the known diversity of Delaware planthoppers (excluding Delphacidae) from 7 to 62, plus provides new state records for MD (22), NJ (5), PA (8) and DC (21) providing species counts for those states as 88, 74, 60 and 46 respectively (Table 2). The Chao1 estimator suggests an additional 12 species may be found in the state. State-level incidence records of 112 species (Table 2) provides some basis for speculation of which species might be missing from the current inventory, and might be interpreted to suggest that the true diversity of planthoppers in Delaware may be closer to 100 species. A better understanding of the habits and finer-scale distribution patterns would be desirable in order to construct a candidate list of species not yet detected in the Delaware fauna. However, some species detected were not previously known from the region (viz. Aphelonema histrionica, Bothriocera drakei, Bothriocera maculata, Cixius angustatus, Sikaiana harti, Poblicia fuliginosa, and Otiocerus reaumurii), suggesting that the compiled species list may yet be substantially incomplete for the combined states.

In addition to the planthopper fauna reported here, a preliminary inventory of the delphacids of Delaware suggests at least 54 species in the state, although additional taxa are likely to be found before the completion of that inventory.

## Taxonomy

### Cixiidae:

A number of specimens presented taxonomic difficulties. In the Cixiidae, specimens that appeared close to Melanoliarus sablensis differed from that depicted by Mead and Kramer (1982: 474) by having an additional ventral process on the aedeagus and a differing arrangement (size and orientation) of the other ventral processes. Similar specimens were observed in the Great Smoky Mountains National Park (Gonzon et al. 2007). In addition to the odd specimens, a specimen much more similar to that depicted by Mead and Kramer (1982) was found. While the possibility that these specimens represent an undescribed species should be investigated, we feel it is likely that they simply represent a variant of the more conventional form, and we have treated them as the same species with respect to biodiversity estimation calculations. Also, a group of females of Melanoliarus with uniformly dark wings were separated from others because they appear to represent a species not found among the males; they were excluded from the species counts.

Emeljanov (2001) moved several Nearctic Pentastirini from Melanoliarus to Pentastiridius and Reptalus. Pentastiridius can be separated from the other two genera by having 12 teeth at the apex of the basitarsus, versus 10 or fewer in Melanoliarus and Reptalus; however, the features of Melanoliarus have not been investigated relative to Reptalus and diagnostic features separating these genera have not been defined. It is probable that Melanoliarus as currently defined is not monophyletic.

### Achilidae:

Species of Cixidia were identified primarily using features described by Beirne (1950), whose key emphasized color, particularly that of the face. He admitted that there was “some variation” (Beirne 1950: 186) within taxa, and key color features were often relativistic, making species difficult to distinguish without access to authoritatively identified specimens , particularly in the context of this study Cixidia fusca, Cixidia pallida, Cixidia variegata, and Cixidia septentrionalis. Unfortunately, Beirne (1950) did not describe sufficient structural features to assist in doubtful cases. A revision of Cixidia would be desirable to address ambiguities, and to describe potential new species from the southwestern US.

### Dictyopharidae:

The only member of Phylloscelis collected by the authors (or the senior author’s students) was Phylloscelis rubra in New Jersey on cranberry (Vaccinium macrocarpon Aiton). This genus is a good example of a taxon that is likely to be in Delaware, but has not yet been found. While there are only 4 species in the genus, and 3 in the study area (Figure 14), the species are best confirmed by genitalic features as presented in McPherson and Wilson (1995).

### Derbidae:

A number of taxonomic issues were found among the Derbidae, including problems separating species in the genera Omolicna, Cedusa and two genera of Otiocerinae (Anotia and Otiocerus). Specimens of Omolicna (Derbidae) could not be definitively identified to species despite there being only 4 described North American species, and only 3 of these eastern - Omolicna fulva (Van Duzee, 1909), Omolicna mcateei (Dozier, 1928), and Omolicna uhleri (Ball, 1902). While literature records suggest that Omolicna uhleri (Ball, 1902) should be the only northern species, it was evident from the genitalia of Delaware specimens that at least 2 species are present. Because the original descriptions are incomplete, and at times conflicting with subsequent authors, we were unable to determine which of the specimens were Omolicna uhleri, and whether the remainder were Omolicna mcateei, Omolicna fulva or undescribed.

The derbid genus Cedusa is diverse and its members require examination of male genitalia for identification, and even then considerable study is required. Two species within this genus were found to differ from the descriptions provided by Flynn and Kramer (1983). Cedusa kedusa bears a large bifid process on both the left and right sides of the aedeagus. For the horizontal ramus of the bifid process on the left side, Flynn and Kramer (1983: 235) state that the apex may be “…occasionally trifurcate and dentate anteapically with the number of teeth varying from none to four…”. Most of the observed specimens in this study had 4–6 teeth, but otherwise agreed with the description of this species. For Cedusa cedusa, a feature in the key (couplet 72) states that this species has the “paramere with inner ventral margin truncately incised in basal portion” (Flynn and Kramer 1983: 135); but for most of our specimens, this feature was rounded or acute. Variations (in this feature and/or details of the processes of the aedeagus) contrast to Flynn and Kramer’s (1983: 228) comment that “all specimens [of Cedusa cedusa] seen are similar to the illustration”, and have led us to consider our specimens as ‘near *cedusa*’ until further evaluation of the variation in this species can be made.

Species in the Otiocerinae tended to be problematic, particularly since most taxa are rare in collections. It is also a problem that otiocerines have been described primarily based on superficial color features whose diagnostic value has not been verified by reference to genitalic features. While attempting to verify our species concepts, we solicited photographs or examined type specimens of select otiocerines. We found that many of the Fitch types (deposited at the USNM) are in poor shape and greatly faded. It is likely that some of the Kirby collection had been lost (see Horn and Kahle, 1935), and 6 of 8 otiocerine Kirby types could not be located at this time (specifically Otiocerus schellenbergii, Otiocerus reaumurii, Otiocerus degeerii, Otiocerus abbotii, Otiocerus coquebertii and Anotia bonnetii [but see below]). It is clear that both Anotia and Otiocerus are in need of revision. The revision should reference genitalic features to verify species identities, provide a critical reexamination of geographic records, and (as needed) designate neotypes for the apparently missing Kirby types, although Kirby (1821) generally provided adequate descriptions. Also, based on Kirby’s (1821) description, it is possible that the *balli* of McAtee (1923) is the same as Kirby’s *schellenbergii*. While McAtee (1923) and Metcalf (1923) may have misapprehended these species, we have retained their view of these taxa until definitive evidence (esp. Kirby’s *schellenbergii* type) can be found.

Ten species of Anotia are reported from the United States (including species formerly in Amalopota Van Duzee, 1889, subsumed under Anotia by Fennah, 1951: 152). Of the 10 species, Anotia caliginosa Ball, 1937, and Anotia lineata Ball, 1937, are southwestern species (recorded from Arizona) and Anotia mcateei (Dozier, 1928), reported from Illinois and Mississippi, does not occur in the study area. Of the remainder, 5 (Anotia  burnetii, Anotia  bonnetii, Anotia  kirkaldyi, Anotia robertsoni, and Anotia westwoodi) are similar in appearance in having white wings whose veins are variably bordered with dark. It is not clear how much intraspecific variation would be expected in features of wing color or pattern, and such patterns were difficult to interpret in the greatly faded Fitch type specimens (we examined types of Anotia robertsonii and Anotia burnetii). Anotia kirkaldyi and Anotia westwoodi share with Anotia bonnetii the presence of dark spots in the apical cells of the forewing, although they may be more prominent in the latter species. Anotia kirkaldyi and Anotia westwoodi can be separated with difficulty based on the presence of darkened wing veins in the former species, but these taxa are otherwise very similar and may not be distinct. Anotia robertsonii is similar to Anotia burnetii in possessing less extensive wing markings than Anotia kirkaldyi, Anotia westwoodi, and Anotia bonnetii; and in possessing dark markings on the dorsum of the abdomen, although in Anotia burnetii the markings are confined to the middorsum of segments 1–3 and in Anotia robertsonii the entire dorsum of subsequent terga (5–7 or 8).

The type specimen of Anotia bonnetii (the type species of the genus) was also sought, along with types of other otiocerines described by Kirby (1821). Kirby (1821) specified that he had a single Anotia bonnetii specimen, which he described and illustrated. The specimen photographed as the type of Anotia bonnetii (at OUMNH) is pinned and spread, missing the abdomen, both wings on the left side, and the head anterior to the eyes; but it was clear that the specimen was not the one used to describe Anotia bonnetii. We feel the type has been mislabelled, and this specimen is actually the type of Otiocerus francilloni. The specimen could readily have been mislabeled when the Oxford Museum type collection was evacuated to the cellar underneath the Ashmolean Museum during World War II. Kirby (1821: 17), reports black spots and bands (“elytris nigro punctatis et fasciatis”) for Otiocerus francilloni, with the black band interrupted, which is consistent with this specimen.

Nine species of Otiocerus are reported from the north of Mexico; two species, Otiocerus abbotii Kirby, 1821, and Otiocerus kirbyii Fitch, 1851; are not reported from the study area (but see below). We examined the types of Otiocerus signoretii and Otiocerus stollii to help confirm features attributed to these species. The type specimen of Otiocerus signoretii, at the USNM, is in rather poor condition, faded, and partially enmeshed in mycelium, but shows the pattern of spots described by Fitch (1856: 394) that was used in subsequent keys to the genus (“…four dots… placed at the angles of an imaginary square…”). Fitch (1856) also reports “…a broad dusky cloud-like stripe from the base to the middle of the inner margin, and extending thence obliquely across to the outer margin at its tip, and sending a very broad branch to the tip of the inner margin…”. In the type specimen, these marking are very faint. The type specimen of Otiocerus stollii Kirby (at OUMNH) consists of only of one front and one hind wing (evidently of the right side), but the forewing was consistent with our understanding of that species.

McAtee (1923: 47) noted within his key that Otiocerus reaumurii, Otiocerus wolfii, and Otiocerus signoretii “may be one species”. While we are confident that Otiocerus wolfii is distinct from the other taxa, Otiocerus reaumurii and Otiocerus signoretii are very similar. Because the type specimen of Otiocerus signoretii is greatly faded, we attempted to diagnose this species from Otiocerus reaumurii by the distribution of dark spots on the wing, in particular the presence of a spot in the costal cell of Otiocerus signoretii. From the available material, these species appear to differ externally mainly in the spot organization. McAtee (1923: 46–47) noted that between the two species, the vitta of Otiocerus reaumurii was broader and ‘percurrent’, and the vitta of Otiocerus signoretii was ‘forked at apex of clavus’, but we have been unable to verify these features. These species are both similar to Otiocerus stollii except for more extensive dark markings of Otiocerus stollii. Interestingly, all observed specimens of Otiocerus reaumurii and Otiocerus signoretii were female, and all observed Otiocerus stollii were male, possibly suggesting that all these species are part of a single sexually dimorphic species. However, we did not observe a sufficient number of specimens to exclude the possibility that this sex ratio was obtained by chance alone. Also, Fitch reported the type of Otiocerus signoretii to be a male, but the condition of the type specimen makes this difficult to confirm.

A single specimen of Otiocerus from Maryland was not clearly associated with any of the described species. The specimen is uniformly pale, head without markings, forewings without spots and with a very faint band. A similar specimen was found among undetermined Derbidae at the USNM. It is possible that this specimen is Otiocerus kirbyii, but we were unable to confirm this identification.

### Flatidae:

The genus Cyarda is under revision by S. Wilson (S. Wilson, pers. comm.). Species in this genus are largely Caribbean. Four Cyarda have been reported from the United States: Cyarda acuminipennis (Spinola, 1839), Cyarda melichari Van Duzee, 1907, Cyarda sordida Fennah, 1965 ( = C. sp. nr. *acutissima* Metcalf & Bruner, 1948; see Fennah, 1965: 115) and Cyarda walkeri Metcalf, 1923. However, Fennah (1965: 112) noted that for Cyarda walkeri it “…must be assumed that this species occurs only in Jamaica”. Metcalf (1923) reported Cyarda acuminipennis from the eastern US, and later from Florida by Metcalf and Bruner (1948), but occurrence of this species in the US has not been subsequently substantiated (e.g., by Fennah 1965). Of the remaining species, Cyarda sordida is reported only from Florida (Fennah 1965) and Cyarda melichari is widely reported in the eastern United States (including the District of Columbia), but its genitalic features have not been compared to the other US species, so it cannot be assumed that Cyarda found outside of Florida (including the D.C. record) are Cyarda melichari as has apparently been previously assumed. The image used here (Figure 3E) is from an undetermined specimen from Ft. Lauderdale, FL.

### Caliscelidae:

Specimens reported as Bruchomorpha sp. n.were collected at Phillips Landing, Sussex Co., DE (on 3 dates) as well as single specimens from Medford, NJ and Baltimore, MD. Superficially, these specimens are similar to Bruchomorpha dorsata, which has been reported in the Mid-Atlantic region by Dozier (1928), Doering (1939), and Wilson and McPherson (1980a); however, the snout is longer than that described by Doering (1939) for Bruchomorpha dorsata, and females are larger than the reported size range for this species. The specimens are also superficially similar to Bruchomorpha beameri Doering, 1939; a Midwestern species, but the dimensions of the snout and coloration of the legs do not match. Unlike both Bruchomorpha beameri and Bruchomorpha dorsata, the aedeagus bears no dorsally directed process, and the ventral process is strongly retrosely curved. We conclude this taxon to be an undescribed species, which will be described after further review of Bruchomorpha species,

### Seasonality

Seasonality data were compiled from available Delaware specimens as a way to begin to understand the life history of local planthopper taxa. From the available seasonality information, it appears that all non-delphacid planthoppers have a single generation a year in Delaware, with the possible exceptions of Bruchomorpha oculata, Aphelonema simplex, and Cixius nervosus. This would be in general agreement with Nickel and Remane (2002) who report that all non-delphacid planthoppers in Germany have a single generation a year. From these data it is evident that Apache degeerii overwinters as an adult, and based on April records that at least Bothriocera cognita and Melanoliarus placitus may overwinter as nymphs. Of the remaining species little can be determined concerning overwintering stage. Nickel (2003) reports that 18.6% of Fulgoromorpha (including Delphacidae) in Germany overwinter as eggs, 61.4% as nymphs, and12.4% as adults, with the remainder unclear. Published literature reports that Flatormenis chloris, Metcalfa pruinosa, Ormenoides venusta, Acanalonia bivittata, Acanalonia conica, Thionia elliptica and Phylloscelis pallescens overwinter as eggs (Wilson and McPherson 1981a, b; Wilson and Wheeler 1987, McPherson and Wilson 1996). Nickel and Remane (2002) report for the German fauna that all cixiids and achilids overwinter as nymphs.

A large number of Melanoliarus placitus were collected in early July of 2002 by the senior author and several students. The series was collected at mercury vapor lights (many specimens landed on trees near the lights instead of at the lights). Interestingly, this time period fell between the last quarter (July 2, 2002) and the New Moon (July 10 2002), which is similar to observations made by Bartlett and colleagues (2008) concerning Membracidae, where large numbers were collected at lights at times near a new moon.

## Conclusion

While the planthoppers of the eastern United States may be characterized as relatively well known from a taxonomic perspective, their faunistics and ecology remain poorly understood. Although Delaware is near the two largest insect collections in the US (the USNM and the American Museum of Natural History, both of which employ hemipterists), it is a testament to our inchoate understanding of US planthopper faunistics that this study has increased our known Delaware fauna by over 700%. The diversity of planthopper species in Delaware is expected to be relatively modest relative to other states because it is small and physiographically rather uniform, and because planthopper diversity tends to generally increase inversely with latitude (and within North America, is greatest overall in the southwest). Here we also report totals of 88 species for Maryland, 74 for New Jersey, 60 for Pennsylvania, and 46 for the District of Columbia based on a compilation of literature records and available specimens. The only other state with a modern, relatively complete, survey of its planthopper fauna is Illinois (Wilson and McPherson 1980b), which reported 150 species, of which 66 were delphacids. In comparison, the total North American planthopper fauna appears to be 12 families, 165 genera and 935 species, of which 61 genera and 338 species are delphacids, and approximately 2/3 of all US planthopper species are western (unpublished data from species checklist compiled by S. W. Wilson, L. B. O’Brien, and C. R. Bartlett). Clearly our understanding of the faunistics of US planthoppers is limited, and our appreciation of planthopper ecology remains in its infancy. Further regional investigations would be helpful in gaining a more complete understanding of the US planthopper fauna.
